# Long noncoding RNA FIRRE contributes to the proliferation and glycolysis of hepatocellular carcinoma cells by enhancing PFKFB4 expression

**DOI:** 10.7150/jca.58097

**Published:** 2021-05-13

**Authors:** Cunyi Shen, Lu Ding, Huanye Mo, Runkun Liu, Qiuran Xu, Kangsheng Tu

**Affiliations:** 1 Department of Hepatobiliary Surgery, The First Affiliated Hospital of Xi'an Jiaotong University, Xi'an 710061, China.; 2 Department of Anesthesiology, The First Affiliated Hospital of Xi'an Jiaotong University, Xi'an 710061, China.; 3 The Key Laboratory of Tumor Molecular Diagnosis and Individualized Medicine of Zhejiang Province, Zhejiang Provincial People's Hospital, Affiliated People's Hospital, Hangzhou Medical College, Hangzhou 310014, China.

**Keywords:** hepatocellular carcinoma, FIRRE, PFKFB4, glycolysis, tumor progression

## Abstract

Recent reports show that long noncoding RNA (lncRNA) FIRRE contributes to the proliferation, apoptosis resistance, and invasion of colorectal cancer and diffuse large B-cell lymphoma. However, the biological function of FIRRE in hepatocellular carcinoma (HCC) remains unknown. Here, we disclosed that the FIRRE level was frequently increased in HCC compared to nontumor tissues. Compared with normal liver cells, we also confirmed the upregulated level of FIRRE in HCC cells. Notably, the FIRRE high expression was related to malignant clinical features, including advanced TNM stage and tumor size ≥5 cm, and conferred to worse survival of HCC. Functionally, FIRRE knockdown repressed the proliferation and glycolysis of HCCLM3 cells. Overexpression of FIRRE strengthened Huh7 cell proliferation and glycolysis. Notably, FIRRE positively regulated the glycolic enzyme 6-phosphofructo-2-kinase/fructose-2,6-biphosphatase 4 (PFKFB4) expression in HCC cells. PFKFB4 was highly expressed and positively associated with FIRRE level in HCC tissues. The upregulated expression of PFKFB4 was associated with high tumor grade and advanced TNM stage. TCGA data revealed that the PFKFB4 high expression indicated a poor prognosis of HCC. Mechanistically, modulating FIRRE level did not affect the stability of PFKFB4 mRNA. FIRRE was mainly distributed in HCC cells' nucleus and promoted PFKFB4 transcription and expression via cAMP-responsive element-binding protein (CREB). PFKFB4 could abolish the effects of FIRRE knockdown on HCC cell proliferation and glycolysis. To conclude, the highly expressed FIRRE facilitated HCC cell proliferation and glycolysis by enhancing CREB-mediated PFKFB4 transcription and expression.

## Introduction

Hepatocellular carcinoma (HCC), one of the most common malignancies, accounts for 85%-90% of primary liver cancer and one of the leading causes of cancer-related deaths worldwide 1. Though the traditional therapeutic strategies have been greatly progressed, and new targeted drugs are frequently discovered, the long-term survival of HCC patients is unsatisfactorily improved 2. Thus, it is necessary to develop novel effective therapeutic targets by exploring the mechanisms of hepatocarcinogenesis.

Transcripts with no apparent protein-coding potential, which are larger than 200 nucleotides, are defined as long noncoding RNAs (lncRNAs) 3. Increasing evidence has demonstrated that lncRNAs play essential roles in the initiation and progression of human cancers via regulating several cellular behaviors, including proliferation, apoptosis, migration, invasion, and glycolysis 4, 5. Our group has previously identified several HCC-related lncRNAs. For instance, we demonstrate lncRNA MCM3AP-AS1 as a tumor-promoting factor in HCC, and it promotes cancer cell growth by sponging miR-194-5p to enhance forkhead box A1 (FOXA1) expression 6. LncRNA KTN1-AS1 and DSCR8 are highly expressed in HCC and contribute to tumor progression by acting as molecular sponges for miR-23c and miR-485-5p, respectively 7, 8. Moreover, lncRNA CASC2 acts as a tumor suppressor in the epithelial-to-mesenchymal transition (EMT) and HCC cell invasion 9. FIRRE is a recently identified cancer-associated lncRNA. FIRRE participates in maintaining histone methylation by facilitating the inactive X chromosome to the nucleolus via interacting with the CCCTC-binding factor (CTCF) 10. FIRRE is frequently upregulated in colorectal cancer (CRC) and promotes the proliferation, migration, and invasion of CRC cells 11. Besides, FIRRE is transcriptionally regulated by MYC and contributes to the proliferation and anti-apoptosis of diffuse large B-cell lymphoma (DLBCL) cells via the Wnt/β-catenin pathway 12. FIRRE is highly expressed in tumor tissues and is markedly associated with neuroblastoma patients' overall survival 13. A recently published paper reports that a four-lncRNA model, including FIRRE, LUCAT1, DDX11-AS1, and LINC01116, effectively predicts the prognosis of HCC 14. However, no study has investigated the biological function of FIRRE in HCC.

Here, we investigated the expression level of FIRRE between HCC tissues and adjacent nontumor tissues and then determined the clinical significance of FIRRE. Furthermore, gain-of and loss-of-function assays were performed to confirm the biological role of FIRRE in HCC cell proliferation and glycolysis. Finally, we discovered the potential mechanism involved in the action of FIRRE in HCC.

## Material and methods

### Clinical samples

Eighty cases of pathologically confirmed HCC tissues and adjacent noncancerous tissues were obtained from patients after signing written informed consent at the 1^st^ Affiliated Hospital of Xi'an Jiaotong University. The participants did not receive radiofrequency ablation, transcatheter arterial chemoembolization, oral targeted drugs, and biological immunotherapy. The tissue samples were maintained in liquid nitrogen for further analysis. The detailed clinical parameters of HCC patients were listed in Table 1. The Ethics Committee of the 1^st^ Affiliated Hospital of Xi'an Jiaotong University approved our study.

### Cell culture and transfection

The human HCC cell lines (HepG2, Huh7, SK-HEP-1, and Hep3B) and a normal hepatic cell line (L02) were purchased from the Cell Bank of Type Culture Collection of the Chinese Academy of Sciences (Shanghai, China) and preserved in our lab 15. The expression vectors (OE-FIRRE and OE-PFKFB4) were constructed by inserting the cDNA products of FIRRE and 6-phosphofructo-2-kinase/fructose-2,6-biphosphatase 4 (PFKFB4) into pcDNA™3.1 (+) Mammalian Expression Vector (Thermo Fisher Scientific, Carlsbad, CA, USA). FIRRE shRNAs (shFIRRE-1 and shFIRRE-2), cAMP-responsive element-binding protein (CREB) shRNA (shCREB), and nontargeting shRNA (shNT) were provided by GenePharma (Shanghai, China). The above vectors were transfected into HCC cells using Effectene® Transfection Reagent (QIAGEN, Hilden, Germany).

### Quantitative real-time PCR (qRT-PCR)

Total RNA of tissue samples or cells was extracted using TRIzol™ Reagent (Thermo Fisher Scientific) and reverse-transcribed to cDNA using the PrimeScript™ RT reagent Kit (Takara, Dalian, China). The qRT-PCR was carried out with the Takara SYBR Green PCR Kit with a CFX96 Touch™ real-time PCR detection system (Bio-Rad Laboratories, Hercules, CA, USA) following the manufacturer's recommendation. β-actin was used as an endogenous control. The 2^-ΔΔCT^ method was carried out to analyze the relative changes in gene expression. Supplementary Table 1 contained all used primers.

### Cell proliferation assay

The viability of HCC cells was detected using the Cell Counting Kit-8 (CCK-8) assay, according to the previously described protocol 16. We used a microplate reader (Multiskan™ FC, Thermo Fisher Scientific) to measure the absorbance at 450 nm. The proliferation of HCC cells was detected by the 5-ethynyl-2′-deoxyuridine (EdU) assay using the Cell-Light™ EdU Apollo®488 *In vitro* Imaging Kit (RIBOBIO, Guangzhou, China) as previously described 16.

### Glycolysis analysis

Pyruvate production, lactate production, and glucose consumption in HCC cells were determined using the pyruvate assay kit (ab65342, Abcam, Cambridge, MA, USA), lactate assay kit (ab65331, Abcam), and glucose assay kit (ab136955, Abcam), respectively, according to the manufacturer's protocol.

### Western blot

Protein extraction and concentration measurements were performed using RIPA lysis buffer (Beyotime, Shanghai, China) and the Bradford protein assay kit (Beyotime), respectively. The protein samples (10-15 μg) were separated by 10% SDS-PAGE and transferred to the PVDF membranes (Millipore, Bedford, MA, USA). Then, the membranes were incubated with 5% skimmed milk and probed with primary antibodies, including PFKFB4 (1:1000, ab137785; Abcam, Cambridge, MA, USA) and CREB (1:1000, 9197s; Cell Signaling Technology, Danvers, MA, USA), overnight at 4 °C. The next day, the membranes were probed with HRP-linked secondary antibodies (Beyotime) and subsequently visualized using an ECL reagent (Millipore). β-actin (1:1000, sc-47778; Santa Cruz Biotechnology, Dallas, TX, USA) was used as the loading control. Reactive bands were captured by the Amersham Imager 680 machine (GE Healthcare Life Sciences, Pittsburgh, PA, USA) and quantified with ImageJ software (1.51k; National Institutes of Health, Bethesda, MD, USA).

### Statistical analysis

Data were presented as the means ± SD (standard deviation) from at least three individual repeats. Statistical comparisons between groups were analyzed by Student's *t*-test and ANOVA using GraphPad Prism 8.0 (GraphPad Inc., San Diego, CA, USA). We analyzed the prognostic values of FIRRE and PFKFB4 using Kaplan-Meier analysis and Log-rank test. Pearson's correlation coefficient was performed to confirm the correlation between FIRRE and PFKFB4 mRNA in HCC tissues. Statistical significance was defined as P<0.05.

## Results

### FIRRE is overexpressed in HCC and confers to poor prognosis

**Figure 1:**
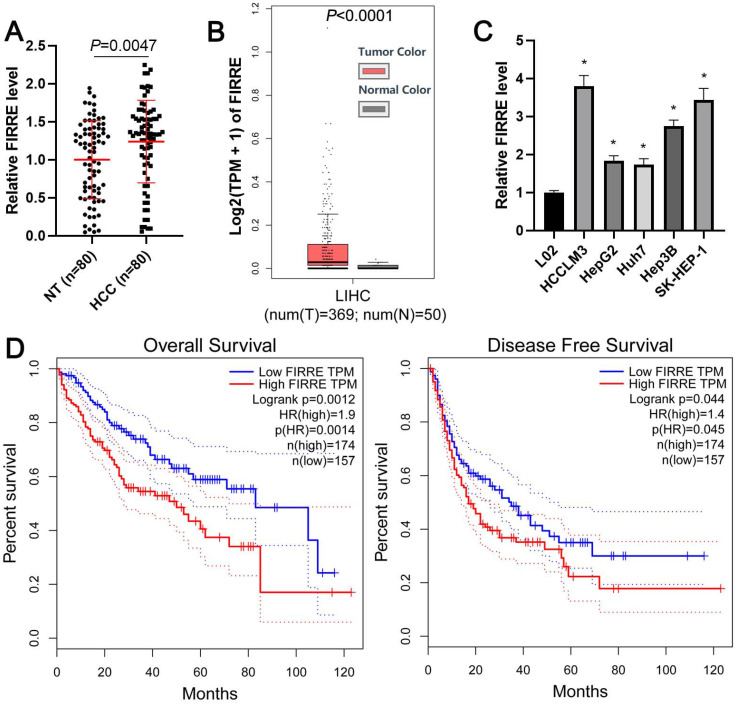
** The overexpression of FIRRE is correlated with the poor prognosis of HCC.** (A) The expression of FIRRE in eighty pairs of HCC tissues and adjacent non-tumor tissues (NT) was assessed by qRT-PCR analysis. (B) TCGA data from the GEPIA website showed an upregulated expression of FIRRE in HCC tissues. (C) The expression levels of FIRRE in the normal hepatic cell line and five HCC cell lines. (D) TCGA data from the GEPIA website demonstrated that the high FIRRE expression indicated poor overall survival and disease-free survival of HCC patients. *P<0.05.

The expression level of FIRRE between HCC and adjacent nontumor tissues was determined using qRT-PCR. As shown in Figure 1A, the expression of FIRRE in HCC was significantly higher than that in tumor-adjacent tissues (P=0.0047). TCGA data from the gene expression profiling interactive analysis (GEPIA) website 17 and GEO data (GSE55092) from the lnCAR website 18 further confirmed the overexpression of FIRRE in HCC compared to normal liver tissues (P<0.001, Figure 1B and Supplementary Figure 1A). Then, we found that the expression of FIRRE in HCC cells (HCCLM3, HepG2, Huh7, Hep3B, and SK-HEP-1) was consistently higher than its expression in immortalized normal liver cells (L02) (P<0.05, Figure 1C). The FIRRE high expression was closely associated with tumor size ≥5 cm (P=0.017) and advanced TNM stage (P=0.014, Table 1). TCGA data analysis using the GEPIA website demonstrated that HCC patients with high FIRRE levels had a prominently poorer overall survival and disease-free survival than those with low FIRRE levels (P<0.05, Figure 1D). Our results indicated FIRRE as an oncogene in HCC.

### FIRRE facilitates the proliferation and glycolysis of HCC cells

**Figure 2:**
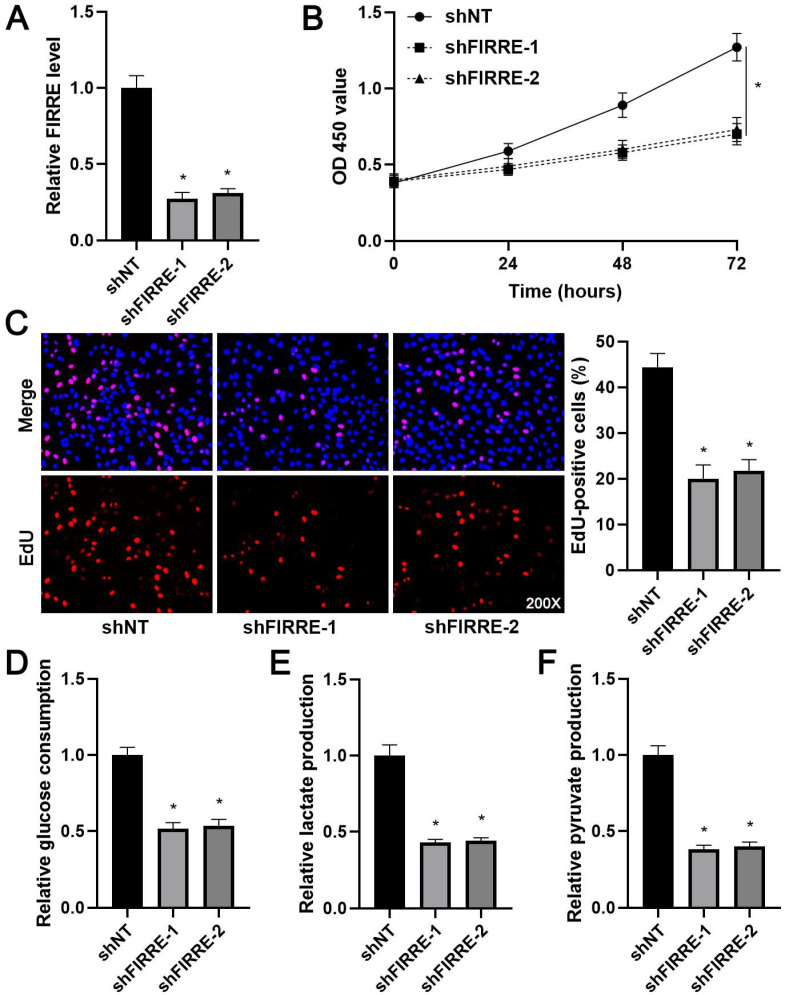
** FIRRE knockdown represses the proliferation and glycolysis of HCCLM3 cells.** (A) shNT or shFIRRE-1/2 was transfected into HCCLM3 cells, and qRT-PCR was performed to analyze FIRRE expression. (B and C) CCK-8 and EdU assays revealed that FIRRE silencing inhibited the proliferation of HCC cells. Original magnification: 200×. (D-F) Silencing of FIRRE decreased glucose consumption, pyruvate production, and lactate production of HCC cells. *P<0.05.

**Figure 3:**
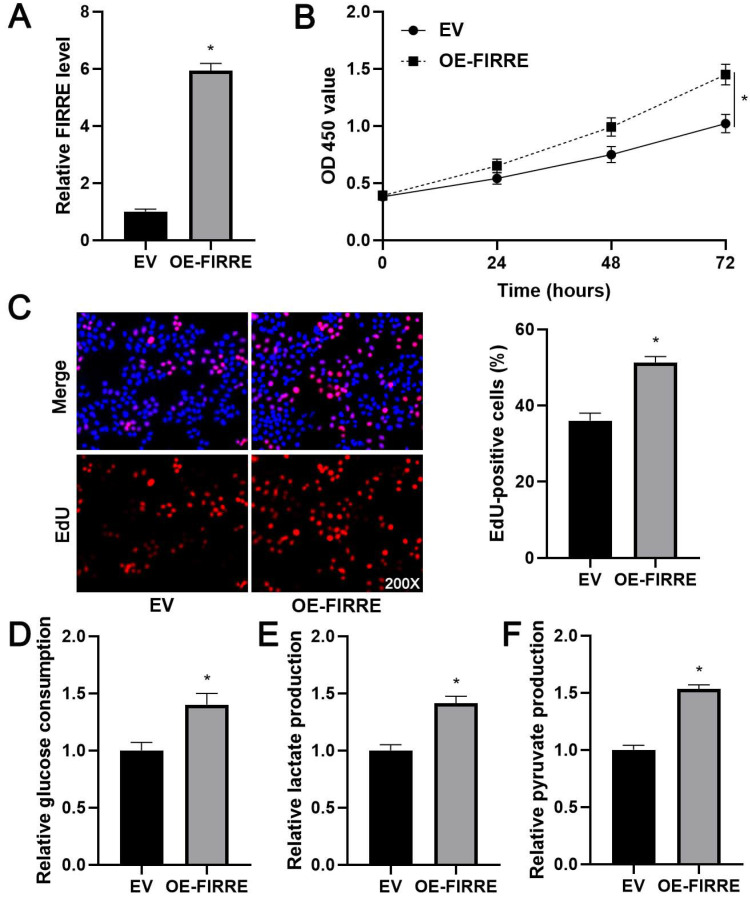
** FIRRE facilitates Huh7 cell proliferation and glycolysis.** (A) OE-FIRRE or empty vector (EV) was transfected into Huh7 cells, and qRT-PCR was carried out to detect FIRRE expression. (B and C) CCK-8 and EdU assays indicated that ectopic expression of FIRRE promoted the proliferation of HCC cells. Original magnification: 200×. (D-F) Ectopic expression of FIRRE promoted glucose consumption, pyruvate production, and lactate production of HCC cells. *P<0.05.

As shown in Supplementary Figure 1B, KEGG pathway analysis of GEO data (GSE55092) indicated that FIRRE was associated with glycolysis. Next, we investigated the effects of FIRRE on HCC cell proliferation and glycolysis. FIRRE expression was down-regulated using two independent siRNA constructs in HCCLM3 cells, which expressed a higher level of FIRRE among tested HCC cell lines (P<0.05, Figure 2A). The FIRRE knockdown prominently repressed the proliferation of HCCLM3 cells as suggested by CCK-8 and EdU assays (P<0.05, Figure 2B and 2C). Moreover, FIRRE silencing prominently reduced glucose consumption, pyruvate production, and lactate production in HCCLM3 cells (P<0.05, Figure 2D-2F). Conversely, FIRRE expression was upregulated by transfecting an expression plasmid in Huh7 cells, which expressed a lower level of FIRRE in tested HCC cell lines (P<0.05, Figure 3A). Our results showed that ectopic expression of FIRRE markedly strengthened the proliferation and glycolic activities of Huh7 cells (P<0.05, Figure 3B-3F).

### FIRRE enhances PFKFB4 expression in HCC cells

**Figure 4:**
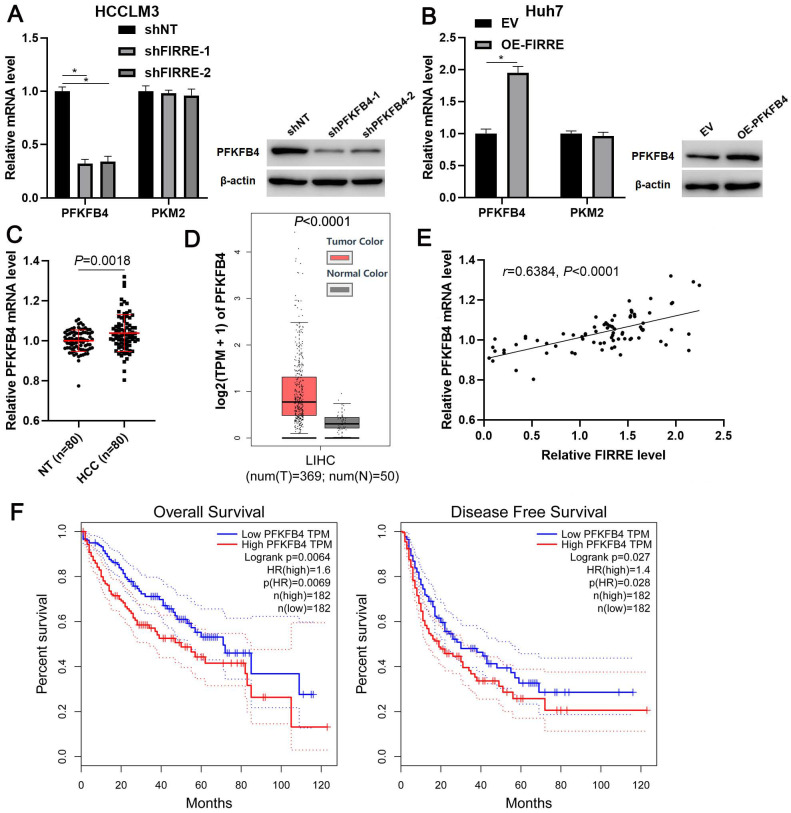
** FIRRE positively regulates PFKFB4 expression in HCC cells.** (A) shNT or shFIRRE-1/2 was transfected into HCCLM3 cells, and qRT-PCR and immunoblotting were performed to analyze PFKFB4 and PKM2 expression. (B) OE-FIRRE or empty vector (EV) was transfected into Huh7 cells, and qRT-PCR and immunoblotting were carried out to detect PFKFB4 and PKM2 expression. (C) The expression of PFKFB4 mRNA in eighty pairs of HCC and adjacent nontumor tissues (NT) was assessed by qRT-PCR analysis. (D) TCGA data from the GEPIA website indicated the upregulated expression of PFKFB4 mRNA in HCC tissues. (E) A positive correlation between FIRRE and PFKFB4 mRNA level was observed in HCC tissues. (F) TCGA data from the GEPIA website demonstrated that the high PFKFB4 expression indicated poor overall survival and disease-free survival of HCC patients. *P<0.05.

Since glycolic metabolism of HCC cells is controlled by several key rate-limiting enzymes, including hexokinase 2 (HK2), PFKFB3, PFKFB4, phosphofructokinase, liver type (PFKL), and pyruvate kinase M2 (PKM2) 19. TCGA data analysis indicated that FIRRE was positively correlated with these enzymes' expression, especially PFKFB4 and PKM2 (P<0.01, Supplementary Figure 2). Thus, we aimed to study the regulatory effects of FIRRE on PFKFB4 and PKM2 levels in HCC cells. We found that FIRRE knockdown markedly reduced PFKFB4 mRNA level in HCCLM3 cells, while FIRRE overexpression markedly increased PFKFB4 mRNA expression in Huh7 cells (P<0.05, Figure 4A and 4B). However, modulating FIRRE level did not impact PKM2 mRNA level in HCC cells (Figure 4A and 4B). Then, western blot results confirmed that FIRRE positively regulated PFKFB4 protein in HCC cells (Figure 4A and 4B). Next, the aberrant expression of PFKFB4 was detected in our HCC cohort. qRT-PCR data confirmed a significantly upregulated level of PFKFB4 mRNA in HCC compared to adjacent nontumor tissues (P=0.0018, Figure 4C). The overexpression of PFKFB4 mRNA in HCC was also observed in TCGA data (P<0.0001, Figure 4D). The upregulated expression of PFKFB4 protein was detected in HCC tissues compared to adjacent nontumor tissues (Supplementary Figure 3). The positive correlation between FIRRE and PFKFB4 mRNA level was detected in our HCC samples (r=0.6384, P<0.0001, Figure 4E). The upregulated expression of PFKFB4 was associated with high tumor grade (P=0.026) and advanced TNM stage (P=0.003, Supplementary Table 2). Importantly, TCGA data analysis based on the GEPIA website revealed that HCC patients with high PFKFB4 mRNA levels showed a significantly worse overall survival and disease-free survival than those with low PFKFB4 mRNA levels (P<0.05, Figure 4F). Our results indicated that FIRRE positive regulated PFKFB4 expression in HCC.

### FIRRE promotes PFKFB4 transcription via CREB

**Figure 5:**
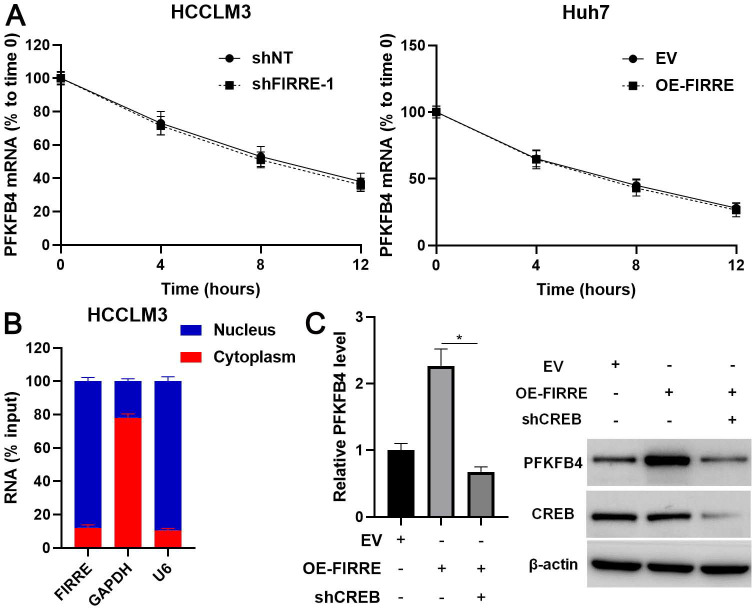
** FIRRE promotes PFKFB4 transcription via CREB in HCC cells.** (A) HCCLM3 and Huh7 cells transfected with indicated vectors were treated with actinomycin D (5 µg/ml) to block transcription. Modulating FIRRE4 expression did not affect PFKFB4 mRNA stability. (B) The cellular distribution of FIRRE, GAPDH, and U6 in HCCLM3 cells. (C) Empty vector (EV), OE-FIRRE, and OE-FIRRE + shCREB were respectively transfected into Huh7 cells. CREB knockdown abolished FIRRE-induced PFKFB4 expression. *P<0.05.

A previous study suggests that FIRRE regulates inflammatory gene expression by enhancing mRNAs' stability 20. Thus, we explored the role of FIRRE in PFKFB4 mRNA stability in HCC cells. However, neither the knockdown of FIRRE nor FIRRE overexpression impacted the stability of PFKFB4 mRNA in HCC cells (Figure 5A). Then, we determined the cellular location of FIRRE and revealed that FIRRE was mainly distributed in the nucleus of HCCLM3 cells (Figure 5B). Thus, we speculated that FIRRE might affect PFKFB4 transcription. Hypoxia-inducible factor-1α (HIF-1α) and CREB have been recognized as transcription factors to control PFKFB4 expression 21-23. HIF-1α mainly exerts its transcription activity under hypoxic conditions. We reduced CREB expression using an shRNA construct in FIRRE overexpressing Huh7 cells (Figure 5C). As expected, CREB knockdown remarkably decreased FIRRE-induced PFKFB4 transcription and expression in HCC cells (P<0.05, Figure 5C). Thus, CREB participated in the regulation of PFKFB4 by FIRRE in HCC cells.

### PFKFB4 mediates the role of FIRRE in HCC cells

**Figure 6:**
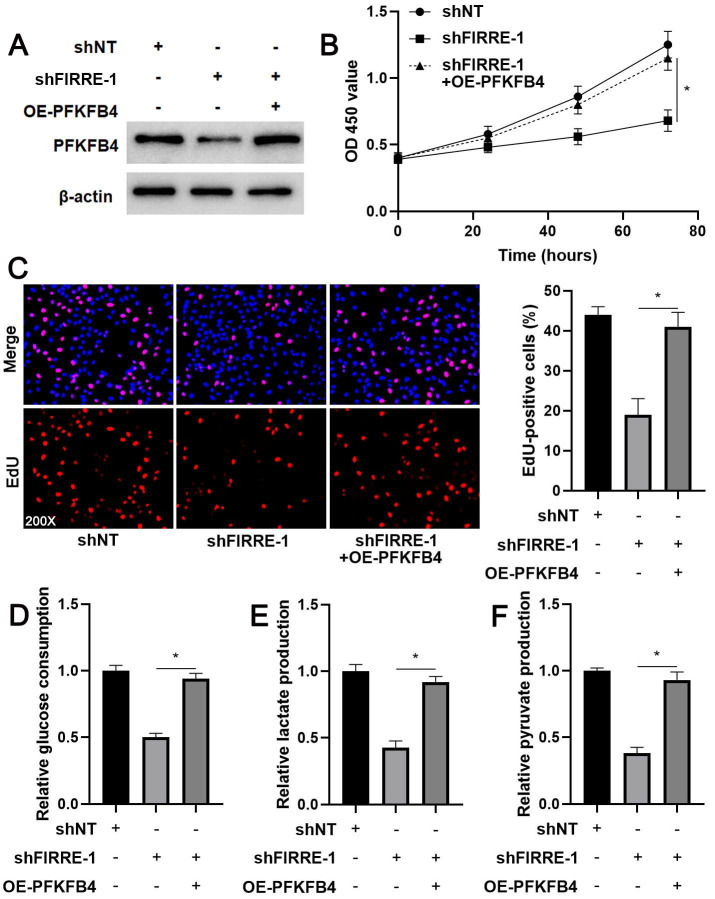
**PFKFB4 abolished the effects of FIRRE knockdown on HCCLM3 cells**. (A) shNT, shFIRRE, and shFIRRE + OE-PFKFB4 were respectively transfected into HCCLM3 cells, and western blotting was performed to detect PFKFB4 expression. (B-F) PFKFB4 overexpression abolished FIRRE knockdown-induced inhibitory effects on the proliferation and glycolysis of HCCLM3 cells. Original magnification: 200×. *P<0.05.

The prior study shows that PFKFB4 knockdown inhibits HCC cell proliferation and glycolysis 24, consistent with FIRRE silencing. Thus, we aimed to determine whether PFKFB4 was a downstream effector of FIRRE in HCC cells. We restored PFKFB4 expression in FIRRE knockdown HCCLM3 cells (Figure 6A). PFKFB4 restoration reversed FIRRE knockdown-induced proliferation arrest in HCCLM3 cells (P<0.05, Figure 6B and 6C). PFKFB4 re-expression enhanced glucose consumption, pyruvate production, and lactate production in FIRRE silenced HCCLM3 cells (P<0.05, Figure 6D-6F). Altogether, our results suggested that FIRRE promoted HCC progression by regulating PFKFB4.

## Discussion

The dysregulation and dysfunction of lncRNAs participate in the initiation and progression of HCC 25. For example, lncRNA LINC01123 is overexpressed in HCC, and its overexpression predicts poor prognosis of patients 16. LINC01123 facilitates the proliferation, migration, and invasion of HCC cells via miR-34a-5p/TUFT1 pathway 16. LncRNA A1BG-AS1 is a tumor-suppressive factor in HCC and functions as a miR-216a-5p sponge to suppress cancer cells' proliferation and invasion 26. LncRNA LINC01554 represses glucose metabolism reprogramming and tumorigenicity of HCC by enhancing the ubiquitin proteolysis of PKM2 and inactivating Akt/mTOR pathway 27. In the current study, we revealed that FIRRE was frequently overexpressed in HCC. We also observed the elevated level of FIRRE in HCC cells. Importantly, TCGA data showed that HCC patients with high FIRRE levels had a significantly shorter overall survival and disease-free survival. The functional experiments indicated that FIRRE promoted the proliferation and glycolic activities of HCC cells. Thus, we identified FIRRE as a novel oncogenic factor in HCC.

Glucose metabolic reprogramming is an essential feature of cancer cells 28. Unlike normal cells, cancer cells prefer to metabolize glucose through glycolysis, thereby enhancing glucose uptake and lactate production 29, 30. This phenomenon is also called the Warburg effect 31. Glycolysis contributes to the growth, apoptosis resistance, metastasis, and immune escape, and drug resistance of HCC 19. There are three rate-limiting enzymes in the glycolysis process, including HK, PFK, and PK 29. Our previous studies have reported the biological role of PKM2 and its underlying mechanisms in HCC progression 32, 33. In this study, we confirmed that FIRRE positively regulated PFKFB4 expression in HCC cells. Studies have shown that lncRNAs not only act as competing endogenous RNA to sponge miRNAs, thereby promoting the expression of miRNAs' target genes, they also interact with cellular macromolecules (such as chromatin DNA, RNA, and protein) 34. Here, we demonstrated the FIRRE increased PFKFB4 expression via promoting gene transcription rather than enhancing mRNA stability in HCC cells. The CREB transcription factor binds to the promoter of PFKFB4 to enhance gene transcription in mouse spermatogenic cells 22. CD44ICD promotes PFKFB4 transcription and expression via interacting with CREB and contributes to the glycolysis and stemness of breast cancer cells 23. Otherwise, CREB is identified as an essential transcription factor in the initiation and progression of HCC 35, 36. Our results showed that CREB knockdown significantly abrogated FIRRE-induced PFKFB4 expression in HCC cells. Thus, we provided a novel insight into the regulator mechanisms involved in PFKFB4 expression in HCC.

Next, we demonstrated that PFKFB4 was highly expressed in HCC as qRT-PCR and western blotting data suggested. Both our data indicated a positive correlation between FIRRE and PFKFB4 mRNA level in HCC tissues. Furthermore, the elevated expression of PFKFB4 mRNA was correlated with malignant clinical features and predicted poor clinical outcomes of HCC patients. Therefore, our data disclosed the clinical value of PFKFB4. A previous study shows that PFKFB4 knockdown represses the proliferation and glycolysis of HCC cells 24. Notably, we found that PFKFB4 restoration abolished FIRRE knockdown-induced effects on the proliferation and glycolysis of HCC cells. Our data suggested that PFKFB4 played a critical role in FIRRE-induced HCC progression.

In summary, we recognized FIRRE as a novel oncogenic factor in HCC. FIRRE enhanced the proliferation and glycolysis of HCC cells by enhancing CREB-mediated PFKFB4 transcription and expression. Both FIRRE and PFKFB4 overexpression correlated with a poor prognosis of HCC. FIRRE may be a potential therapeutic target and prognostic indicator in HCC.

## Supplementary Material

Supplementary figures and tables.

## Figures and Tables

**Table 1:** The correlation between FIRRE expression and clinicopathologic characteristics of hepatocellular carcinoma

Characteristics	n=80	FIRRE expression		*P* (chi-square test)
		Low (n=40)	High (n=40)	
**Age (years)**				
<50	35	19	16	0.499
≥50	45	21	24	
**Sex**				
Male	63	33	30	0.412
Female	17	7	10	
**HBV**				
No	28	18	10	0.061
Yes	52	22	30	
**Serum AFP level (ng/mL)**				
<20	27	16	11	0.237
≥20	53	24	29	
**Tumor size (cm)**				
<5	26	18	8	0.017*
≥5	54	22	32	
**No. of tumor nodules**				
1	65	34	31	0.390
≥2	15	6	9	
**Cirrhosis**				
No	35	21	14	0.115
Yes	45	19	26	
**Venous infiltration**				
No	44	25	19	0.178
Yes	36	15	21	
**Edmondson-Steiner grade**				
I+II	57	31	26	0.217
III+IV	23	9	14	
**TNM stage**				
I+II	63	36	27	0.014*
III+IV	17	4	13	

HBV, hepatitis B virus; AFP, alpha-fetoprotein; TNM, tumor-node-metastasis. The “low” or “high” expression of FIRRE level was defined according to the cut-off value, which was defined as the median value of the cohort of patients tested. *Statistically significant.

## Abbreviations

HCC: hepatocellular carcinoma
lncRNA: long noncoding RNA
FOXA1: forkhead box A1
EMT: epithelial-to-mesenchymal transition
PFKFB3/4: 6-phosphofructo-2-kinase/fructose-2,6-biphosphatase 3/4
CREB: cAMP responsive element binding protein
GEPIA: gene expression profiling interactive analysis
HK2: hexokinase 2
PFKL: phosphofructokinase, liver type
PKM2: pyruvate kinase M2
HIF-1α: hypoxia-inducible factor-1α
