# Supplementary material for: Long noncoding RNA FIRRE contributes to the proliferation and glycolysis of hepatocellular carcinoma cells by enhancing PFKFB4 expression
Source: J Cancer. 2021 May 13;12(13):4099–108. doi: 10.7150/jca.58097 (PMC8176253; doi:10.7150/jca.58097)
Supplement: Supplementary file 1 — Supplementary figures and tables. [file jcav12p4099s1.pdf]

Supplementary Table 1. Primers used in the present study

Primer	Sequence (5′-3′)
FIRRE	Forward: CTGTGACCTCGCTTCACTTCT
	Reverse: GTGGCAAAGAGCAGAAGATAGA
PFKFB4	Forward: GGAGTTCAATGTTGGCCAGT
	Reverse: TCAGGATCCACACAGATGGA
PKM2	Forward: CCGCCGCCTGGCGCCCATTA
	Reverse: CGGTCAGCACAATGACCACATC
β-actin	Forward: TTCCTTCCTGGGCATGGAGT
	Reverse: TACAGGTCTTTGCGGATGTC

Supplementary Table 2. The correlation between PFKFB4 expression and clinicopathologic characteristics of hepatocellular carcinoma

Variable		n	PFKFB4 expression		*P*
			Low (n=40)	High (n=40)	
Age (year)	<50	35	13	12	0.809
	≥50	45	27	28	
Gender	Male	63	29	34	0.172
	Female	17	11	6	
HBV infection	Absent	28	17	11	0.160
	Present	52	23	29	
Serum AFP level (ng/mL)	<20	27	17	10	0.098
	≥20	53	23	30	
Tumor size (cm)	<5	26	16	10	0.152
	≥5	54	24	30	
No. of tumor nodules	1	65	31	34	0.390
	≥2	15	9	6	
Cirrhosis	Absent	35	19	16	0.499
	Present	45	21	24	
Venous infiltration	Absent	44	23	21	0.653
	Present	36	17	19	
Edmondson-Steiner grading	I+II	57	33	24	0.026*
	III+IV	23	7	16	
TNM stage	I+II	63	37	26	0.003*
	III+IV	17	3	14	

HBV, hepatitis B virus; AFP, alpha-fetoprotein; TNM, tumor-node-metastasis.

The "low" or "high" expression of PFKFB4 level was defined according to the cut-off value, which was defined as the median value of the cohort of patients tested.

*Statistically significant.

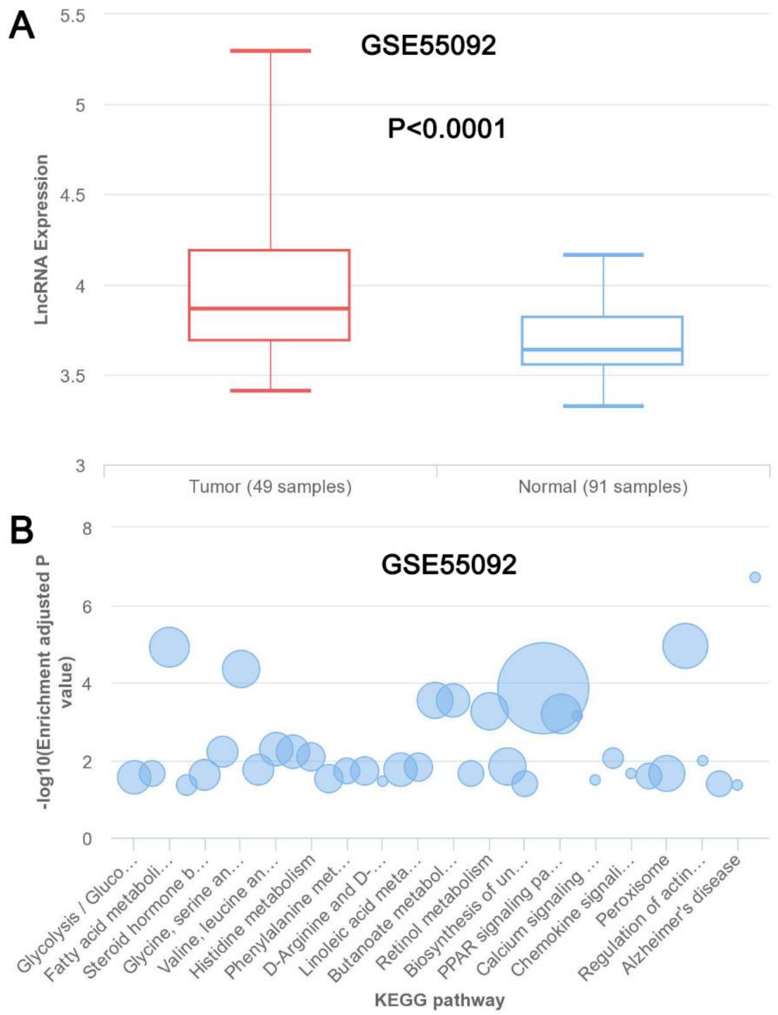

Supplementary Figure 1 The expression and KEGG pathway analysis of FIRRE in HCC using GEO data from lnCAR website. (A) The expression of FIRRE between HCC and normal liver tissues. (B) KEGG pathway analysis of FIRRE based on GSE55092 dataset.

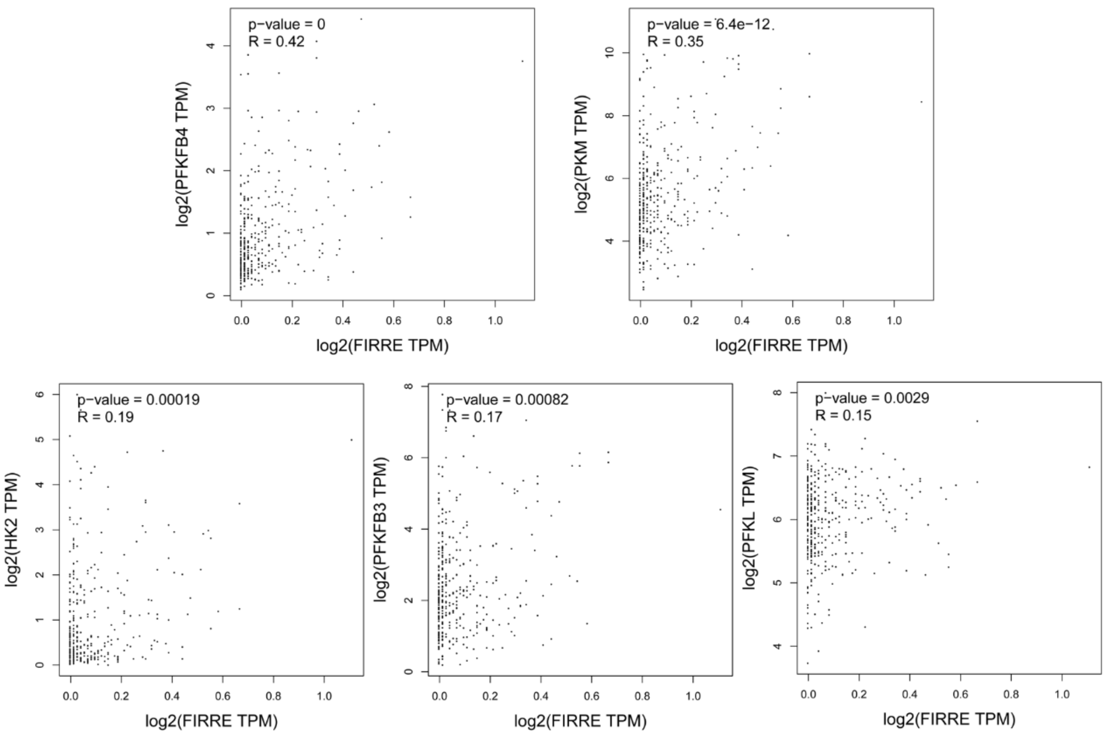

Supplementary Figure 2 The correlations between FIIRE and glycolic enzymes in TGCA-LICH from the GEPIA website.

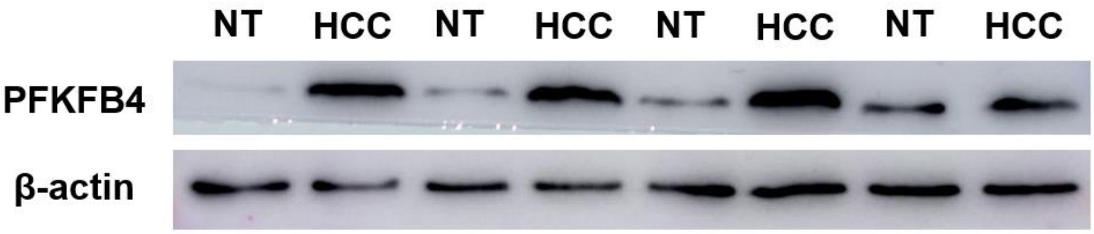

Supplementary Figure 3 The expression of PFKFB4 protein in four pairs of HCC and adjacent nontumor tissues.
